# Enhanced whole genome sequence and annotation of *Clostridium stercorarium* DSM8532^T^ using RNA-seq transcriptomics and high-throughput proteomics

**DOI:** 10.1186/1471-2164-15-567

**Published:** 2014-07-07

**Authors:** John J Schellenberg, Tobin J Verbeke, Peter McQueen, Oleg V Krokhin, Xiangli Zhang, Graham Alvare, Brian Fristensky, Gerhard G Thallinger, Bernard Henrissat, John A Wilkins, David B Levin, Richard Sparling

**Affiliations:** Department of Microbiology, University of Manitoba, Winnipeg, Canada; Manitoba Centre for Proteomics and Systems Biology, University of Manitoba, Winnipeg, Canada; Department of Plant Sciences, University of Manitoba, Winnipeg, Canada; Department of Biosystems Engineering, University of Manitoba, Winnipeg, Canada; Core Facility Bioinformatics, Austrian Centre of Industrial Biotechnology (ACIB), Graz, Austria; Institute for Genomics and Bioinformatics, Graz University of Technology, Graz, Austria; Architecture et Fonction des Macromolécules Biologiques, Université Aix-Marseille, Marseille, France; UMR 7257, Centre National de Recherche Scientifique, 163 ave. de Luminy, Marseille, 13288 France

**Keywords:** Genome, Proteome, Transcriptome, RNA-seq, Tandem mass spectrometry, Proteogenomics, Glycolysis, Pentose phosphate pathway, Transaldolase

## Abstract

**Background:**

Growing interest in cellulolytic clostridia with potential for consolidated biofuels production is mitigated by low conversion of raw substrates to desired end products. Strategies to improve conversion are likely to benefit from emerging techniques to define molecular systems biology of these organisms. *Clostridium stercorarium* DSM8532^T^ is an anaerobic thermophile with demonstrated high ethanol production on cellulose and hemicellulose. Although several lignocellulolytic enzymes in this organism have been well-characterized, details concerning carbohydrate transporters and central metabolism have not been described. Therefore, the goal of this study is to define an improved whole genome sequence (WGS) for this organism using in-depth molecular profiling by RNA-seq transcriptomics and tandem mass spectrometry-based proteomics.

**Results:**

A paired-end Roche/454 WGS assembly was closed through application of an *in silico* algorithm designed to resolve repetitive sequence regions, resulting in a circular replicon with one gap and a region of 2 kilobases with 10 ambiguous bases. RNA-seq transcriptomics resulted in nearly complete coverage of the genome, identifying errors in homopolymer length attributable to 454 sequencing. Peptide sequences resulting from high-throughput tandem mass spectrometry of trypsin-digested protein extracts were mapped to 1,755 annotated proteins (68% of all protein-coding regions). Proteogenomic analysis confirmed the quality of annotation and improvement pipelines, identifying a missing gene and an alternative reading frame. Peptide coverage of genes hypothetically involved in substrate hydrolysis, transport and utilization confirmed multiple pathways for glycolysis, pyruvate conversion and recycling of intermediates. No sequences homologous to transaldolase, a central enzyme in the pentose phosphate pathway, were observed by any method, despite demonstrated growth of this organism on xylose and xylan hemicellulose.

**Conclusions:**

Complementary omics techniques confirm the quality of genome sequence assembly, annotation and error-reporting. Nearly complete genome coverage by RNA-seq likely indicates background DNA in RNA extracts, however these preps resulted in WGS enhancement and transcriptome profiling in a single Illumina run. No detection of transaldolase by any method despite xylose utilization by this organism indicates an alternative pathway for sedoheptulose-7-phosphate degradation. This report combines next-generation omics techniques to elucidate previously undefined features of substrate transport and central metabolism for this organism and its potential for consolidated biofuels production from lignocellulose.

**Electronic supplementary material:**

The online version of this article (doi:10.1186/1471-2164-15-567) contains supplementary material, which is available to authorized users.

## Background

Consolidated bioprocessing (CBP) refers to single-vessel microbial transformation of inexpensive biomass such as agricultural or forestry cellulosic wastes into fuels or other useful chemicals. This approach is based on the power of specific microbes or consortia to simultaneously degrade and transform plant cell wall components into ethanol or other molecules of interest [1, 2]. Cellulolytic clostridia such as *Clostridium thermocellum* and *C. stercorarium* are among the most widely studied organisms for CBP, producing a wide range of cellulases, xylanases and other lignocellulolytic enzymes [3]. However, multiple end products resulting from branching metabolic pathways and low overall ethanol production mitigates the feasibility of industrial CBP using these organisms. For example, selected strains of *C. stercorarium* have been shown to produce up to ~0.4% w/v (80-100 mM) ethanol in laboratory batch cultures [4]. Improvements of at least an order of magnitude will be required to rival current yeast/starch-based processes for bioethanol production.

Two main strategies have emerged to increase ethanol production by these organisms. First, genetic modification has been applied and found to modestly improve yields of ethanol or other biofuels, usually through knocking out elements in undesired metabolic pathways [5, 6]. Second, defined co-cultures of organisms with contrasting or potentially synergistic enzymes for lignocellulose degradation and utilization have been applied, again with modest improvements in biofuels production [7–9]. Central to both of these strategies is a refined conceptual framework and well-defined lignocellulolytic and central metabolic pathways for organisms of interest [10]. To this end, application of next-generation systems biology tools, including genome sequencing and transcriptional/protein profiling has expanded rapidly in the past 10 years [4, 11–13], along with increasingly sophisticated techniques for integrating and visualizing these vast datasets [12, 14, 15]. Basic techniques in this field are in constant flux and yielding ever more detailed information. For example, increasingly powerful mass spectrometers are reducing the importance of gel-based separation or laser desorption techniques in proteomics [12] and microarrays for transcriptomics are increasingly displaced by next-generation RNA sequencing (RNA-seq) [16]. Genome sequencing has become trivial at a technical level, as evidenced by the steady accumulation of brief announcements in the literature, however the currency of in-depth molecular profiling provides an opportunity to improve, confirm and contextualize genome sequence data. This information is critical for designing and interpreting effects of metabolic engineering or co-culture experiments to improve biofuel yields.

With the ultimate goal of developing “designer co-cultures” for biofuels production, our group has recently published genome- and proteome-level descriptions of central metabolism in biofuels organisms of interest [10, 11, 17]. The ethanologenic thermophile *Clostridium stercorarium* DSM8532^T^ has been investigated extensively to characterize its complement of lignocellulolytic enzymes [4, 18–31], however substrate transport and central metabolic pathways for this organism have not been described in detail. Preferential hemicellulolysis and xylose utilization by *C. stercorarium* [3] suggests that it may be compatible in co-culture with *C. thermocellum*, a rapid cellulose-degrader that does not metabolize xylose. Therefore, the goal of this study was to define metabolic potential encoded by the whole genome sequence of *C. stercorarium* DSM8532^T^, in the context of high-throughput molecular profiling using RNA-seq and high-throughput tandem mass spectrometry.

## Methods

### Anaerobic culture

*C. stercorarium* DSM8532^T^ was acquired from Deutsche Sammlung von Mikroorganismen und Zellkulturen (Braunschweig, Germany) and sub-cultured to single colonies on simplified 1191 agar: (w/v) 0.15% potassium phosphate (KH_2_PO_4_), 0.335% sodium phosphate (Na_2_HPO_4_), 0.05% ammonium chloride (NH_4_Cl), 0.018% magnesium chloride (MgCl_2_), 0.1% L-cysteine, 1 ml of 0.025% w/v resazurin solution (all from Sigma-Aldrich, Oakville, Canada), 0.2% yeast extract, and 0.8% agar (both from BD, Mississauga, Canada), pH 7.2. Resuspended stock was spread onto non-reduced plates under normal aerobic conditions in a biosafety cabinet and incubated in jars with GasPak EZ sachets (BD) for 72 h at 65°C. Growth from plates inoculated with single colonies were transferred aseptically to 50 ml liquid culture (as above, except agar) using a syringe and needle in nitrogen-gassed butyl-stoppered serum bottles with cellobiose or xylose (both from Sigma-Aldrich) added aseptically after autoclaving (1 ml of filter-sterilized, degassed 10% solution, final concentration 0.2% w/v). Serial transfers were inoculated with 5 ml (10%) overnight pre-cultures (18–24 h, OD_600_ ~ 0.8). Concentration of hydrogen and carbon dioxide were determined using a Varian benchtop gas chromatograph (Agilent, Mississauga, USA) using standard curves made with degassed butyl-stoppered bottles containing known concentrations of each gas (both from Welders Supplies, Winnipeg, Canada). Concentrations of liquid components (cellobiose, xylose, acetate, lactate and ethanol, all from Sigma-Aldrich) were determined by high-pressure liquid chromatography (HPLC) using an isocratic pump (model #1515) and refractive index detector (model #2414, Waters, Milford, USA), with standard curves derived from stock solutions of each component. Genomic DNA was isolated from overnight cultures growing on 0.2% cellobiose using the Genomic DNA Wizard kit (Promega, Madison, USA) according to supplier’s protocol. RNA for transcriptomics was isolated from mid-exponential phase culture (1 × 50 ml, 12 h, OD_600_ ~ 0.4), on 0.2% cellobiose. Protein was isolated from pooled mid-exponential phase cultures (25 × 50 ml, 12 h, OD_600_ ~ 0.3) on 0.2% xylose, as detailed below.

### Whole-genome sequencing, assembly and annotation

The genome was sequenced using the GS-FLX Titanium platform (Roche/454, Branford, USA) and the resulting 8 kilobase paired-end library of 358,837 reads was assembled using Newbler (v2.6), resulting in 26-fold coverage and 120 contigs, of which 33 were joined by Newbler into a single large scaffold encompassing 96% of total sequence with 33 gaps. Remaining contigs represented repetitive sequences (16S, 23S and different transposons). Gaps resulting from these repetitive sequences were resolved by *in silico* gap filling, where contigs generated from a gap-specific assembly were integrated into the circular scaffold with a custom R (version 2.15.1) [32] script (Thallinger *et al.*, manuscript in preparation). One ambiguous region and one gap remained, with the latter closed by gap edge-specific primer design using Primer-BLAST (http://www.ncbi.nlm.nih.gov/tools/primer-blast) followed by bidirectional Sanger sequencing of resulting amplicon using the ABI3100 Genetic Analyzer (Life Technologies, Burlington, Canada). Origin of replication was determined with originx [33], and the genome was rearranged to start at this position with the chromosomal replication initiator DnaA as the first protein. Raw sequencing reads (.sff file) were submitted to the Sequence Read Archive of NCBI (http://www.ncbi.nlm.nih.gov/sra), with accession SRX481570.

Automated gene-calling and functional annotation (EC number, COG, Pfam, TIGRfam, KEGG, Metacyc) was carried out through submission of sequence assembly to Joint Genome Institute’s Integrated Microbial Genomes Expert Review (IMG/ER) [34]. Lignocellulolytic enzymes were identified and categorized by comparison with the Carbohydrate-Active Enzyme (CAZy) database [35]. Identification and classification of transporters was carried out initially using the Transporter Classification function in IMG/ER, which is based on the TCDB database (http://www.tcdb.org) [36]. The subset of ABC transporters with predicted carbohydrate uptake activity was established through analysis of functional annotations in IMG/ER and further characterized using the ABC transporter database (http://www-abcdb.biotoul.fr) [37]. Functional annotation of well-characterized enzymes was used to create categories for specific metabolic pathways of interest (glycolysis, pentose phosphate pathway, pyruvate/PEP conversion), as previously described [10, 17]. Hydrogenases and other enzymes important in co-factor recycling and energy balance were identified by homology to known clostridial enzymes [10, 17, 38]. Nearest neighbour (top bit score in maximum overlap) was established by BLAST using the NCBI website.

Annotated coding sequences were evaluated through submission of draft assembly to GenePRIMP annotation improvement platform of the Joint Genome Institute (http://geneprimp.jgi-psf.org) [39] to identify long, short, unique, dubious, split or missed genes. Automatically generated annotation information was downloaded from IMG/ER to construct the feature file (.tbl) required for NCBI submission using sort and concatenation functions in Excel. Manual curation of all automatically generated product names was carried out using NCBI instructions (http://www.ncbi.nlm.nih.gov/genbank), with further annotation based on database comparisons and sequence improvements using transcriptomic and proteomic data as described below. The .sqn file for NCBI submission was generated using tbl2asn and adjusted through error-reporting, followed by provisional submissions and further error correction. The final closed circle assembly and annotation was approved by NCBI on January 10, 2013 and first public draft released on March 31, 2013 with accession [GenBank: CP003992]. The RefSeq accession is [GenBank: NC_020887]. In order to independently confirm specific observations regarding whole genome sequence and specific metabolic pathways, an independently-derived whole genome sequence of the same strain [40] was accessed [GenBank: NC_020134].

### RNA-seq transcriptomics and correction of homopolymers

RNA was isolated using the ChargeSwitch magnetic bead-based technique for RNA extraction (Life Technologies, Burlington, Canada), including DNaseI treatment of crude extracts, according to manufacturer’s directions. Briefly, total RNA was quantified using a NanoDrop Spectrophotometer ND-1000 (NanoDrop Technologies, Wilmington, USA) and integrity assessed using a 2100 Bioanalyzer (Agilent Technologies, Mississauga, Canada). The ribosomal RNA depletion was done using 1 μg of total RNA with the Metabacteria Ribo-Zero rRNA Removal Kit (Mandell Scientific, Guelph, Canada). After rRNA depletion, remaining RNA was purified using the RiboMinus Concentration Module (Life Technologies), with final elution in 17 μl of “Fragment, Prime, Finish” mix instead of water, followed by fragmenting and priming for cDNA synthesis. Starting at the “First strand cDNA synthesis” step of the protocol for TruSeq Stranded mRNA Sample Prep Kit (Illumina, San Diego, USA), samples were converted into a library suitable for cluster generation and DNA sequencing. Library quality was assessed using a LabChip and Light Cycler 480 II (Roche, Mississauga, Canada) for size and an Infinite M200 Fluorimeter (Tecan, Mannedorf, Switzerland) for quantification. cDNA transcripts (2 × 100 bp) were sequenced with the Illumina HiSeq 2000 platform by McGill University and Genome Quebec Innovation Center. A total of 2.6 million reads (52 Gb) with an average Phred quality score of 34 (100% passed filter) were sequenced, with an expected false discovery rate (base-calling error) of 0.05% based on the quality control plot. Raw reads (.fastq file) were pre-processed using a custom script incorporating Trimmomatic (http://www.usadellab.org) with default settings (see Additional file 1). This algorithm trims adapters, removes leading or trailing low quality and N bases (Phred score > 3), scans reads in a 4 bp sliding window and cuts when average quality score falls below 15, and removes all reads of less than 36 bp. Tophat [41] was used for read alignment based on the reference genome annotation, genome sequence and paired end insertion information. Final reads with greater than 2 mismatches, gaps or indels were discarded. Coverage of the genome sequence by RNA-seq transcripts was determined by generating a “base pair map” of the .bam alignment using the bam2depth function in SAMtools (http://samtools.sourceforge.net) [42]. Raw Illumina sequencing reads in .fastq format were submitted to the Sequence Read Archive of NCBI (http://www.ncbi.nlm.nih.gov/sra) with accession SRX481592.

Transcriptomic datasets were compared with the genome sequence in order to correct homopolymer errors in 454 pyrosequencing, confirm coding regions and parse improvement suggestions identified through GenePRIMP and NCBI error reporting. For RNA-seq data, individual Illumina reads were mapped to the genome sequence using CLC Genomics Workbench (CLC Bio, Aarhus, Denmark), resulting in a list of sequence variants that were manually checked against the mapping of 454 reads and further validated in reference to a concurrent genome sequence for this organism (GenBank Accession: NC_020134) [40].

### 2D-HPLC MS/MS proteomics and proteogenomics

Protein was extracted from PBS-washed cell pellets by sonication in the presence of detergent, digested using trypsin, cleaned and fractionated as previously described [11, 43, 44]. Resuspended peptide fractions were subjected to two-dimensional HPLC (40 1-minute fractions collected at pH 10, pairwise concatenated into 20 fractions, with 1-hour gradients for each at pH 2 formic acid) coupled to tandem mass spectrometry (MS/MS) [45], using the TripleTOF 5600 platform (AB Sciex, Concord, Canada). Results were concatenated from 20 LC-MS/MS runs of 70 minutes each and converted from native WIFF format to mascot generic format (.mgf) using the Analyst built-in conversion utility. The collision-induced dissociation (CID) spectra in this file were analyzed using X!tandem (2012.10.01.1) against a database of annotated proteins (.fasta format), using the following search settings: fixed modification C + 57.021; parent mass error: + − 20 PPM; fragment mass error + −0.05 Da. Peptides with an expectation value of log(e) < −1 were reported [46]. In order to determine coverage of the genome by MS/MS peptides, a “base pair map” assigning ion current values to the genome sequence was created using a custom Python script (see Additional file 1). Mass spectra (http://hs2.proteome.ca/tandem/archive/csterc2dproteogenomic.mgf.txt) are available at the Manitoba Centre for Proteomics and Systems Biology Global Proteome Machine server.

Proteogenomics analysis was carried out as previously described [17], with the goal of determining whether non-specific hits provide any information about protein coding regions not captured in automatic annotation procedures. An alternative database based on raw 454 reads instead of annotated proteins was created. Quality information was discarded, and each read transcribed across 6 reading frames into peptide-source elements between STOP codons only (no START codon required). Each element was subjected to an *in silico* single-missed cleavage tryptic digestion, resulting in a non-redundant proteogenomic peptide database. This collection of peptides was assembled into .fasta format (http://hs2.proteome.ca/tandem/archive/naive454csterc.fasta) and analyzed using X!tandem as described above. It is important to note that “proteins” in this database have no connection to actual assembled-annotated proteins. Rather, they are collections of connected proteogenomic tryptic peptides for purposes of identifying MS/MS spectra. The results file was parsed into non-redundant member peptides, filtered to exclude peptides containing variable post-translational modifications, and scanned against the genome annotation. Unassigned peptides were analyzed using the TblastN function in IMG/ER in order to assign them to potential source proteins in related organisms.

### Coverage of genome by transcriptome and proteome

Coverage of each locus in the genome by RNA-seq reads and MS/MS peptides was calculated by comparing base pair maps to gene regions using a custom R script (see Additional file 1). This analysis also created .fasta files where each base observed in reads/peptides is itself and each base not observed is represented by a dash. To visualize genome coverage, these .fasta files were uploaded along with the genome record (.gbk file) to the Gview visualization platform (http://www.gview.ca) [15], and rendered using the BLAST atlas function.

## Results

### Whole genome sequence and gene coverage by reads/peptides

**Figure 1:**
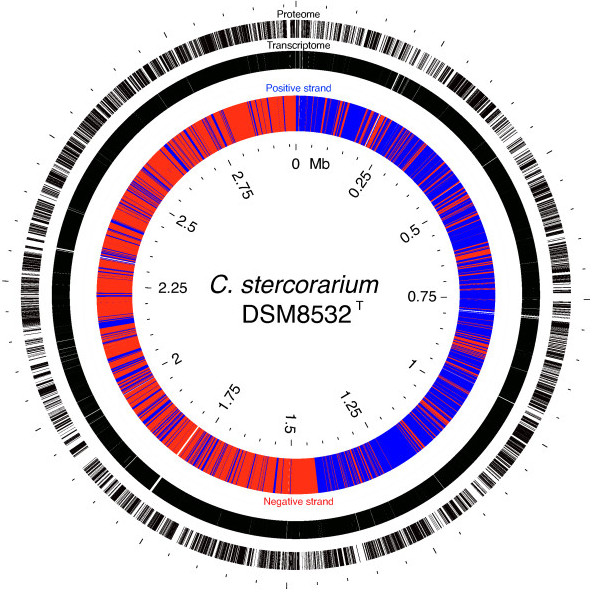
***Complete genome, transcriptome and proteome of C. stercorarium DSM8532*** ^***T***^ **.** Inner ring shows all genes in complete genome (positive strand in blue, negative strand in red). Middle ring shows nearly complete coverage of genes by sequence reads generated by Illumina RNA-seq. Outer ring shows extensive and evenly distributed coverage of coding regions by peptides detected by MS/MS.

A closed genome sequence was generated from a single 454 pyrosequencing run, using a novel *in silico* technique for gap-closing and integration of repetitive regions (Thallinger *et al.*, manuscript in preparation). Wet-lab sequence determination was required for a single gap of 29 base pairs only, with one region of 2 kilobases containing 10 ambiguous bases left unresolved. The final whole genome sequence is 2.97 megabases, with 2,580 protein-coding regions and 61 non-coding (RNA) genes, including 3 ribosomal RNA, 48 transfer RNA genes and 4 miscellaneous RNAs (see Additional file 2: Table S1). Genome sequence coverage by RNA-seq reads and mass spectrometry peptides was extensive. Almost all genes (2575/2641 or 97.5% were completely covered by RNA-seq reads, indicating extensive baseline transcription and/or possible residual DNA in RNA preps (Additional file 2: Table S1). About one-third of protein-coding genes (815/2580 or 32%) had no peptide coverage (Additional file 2: Table S1). Visualization of transcriptomic and proteomic coverage using Gview confirmed even distribution of RNA-seq reads and MS/MS peptides across the entire genome (Figure 1).

### Genome improvement by RNA-seq

A total of 94 alternative coding regions were identified by GenePRIMP and/or NCBI (Additional file 3: Table S2). Assembled RNA-seq reads were used to identify and correct 35 errors in genome sequence data due to inaccurate 454 sequencing of homopolymer stretches (Table 1). Several bases identified by RNA-seq simply contradicted the genome sequence, indicating error by either method or small-scale mutations in working stocks of lab strains. Sequence corrections by RNA-seq corroborated five suggested joins and one suggested extension by GenePRIMP/NCBI (Table 1). Although the remaining differences were not independently confirmed in this study (ie. by Sanger sequencing of contradictory sequence regions), comparison with a recently-published genome sequence [40] confirmed the majority of corrections suggested by RNA-seq (Table 1).

**Table 1:** **Base changes, additions and deletions in** ***C. stercorarium*** **DSM8532** ^**T**^ **genome sequence suggested by RNA-seq transcriptome**

Gene/interval	Former/final start position ^1^	Original	Corrected	Offset ^2^	Corroborated by alternate genome
Clst_0020/21	21928	G	-	−1	Y
Clst_0024	25417	A	G	NA	Y
Clst_0030/1	31758	T	-	−1	Y
Clst_0035/6	37728	-	A	1	Y
Clst_0081	88853	A	T	NA	Y
Clst_0081	88886	C	A	NA	Y
Clst_0130*	**149305**	**-**	**A**	**1**	**Y**
Clst_0131/2	150526	-	A	1	Y
Clst_0269	313700	A	G	0	Y
Clst_0746*	**820762**	**-**	**A**	**1**	**Y**
Clst_0755/6	828349	-	T	1	Y
Clst_0791/2	868841	C	A	0	Y
Clst_0847	937715	-	C	1	Y
Clst_0897/8	995753	-	A	1	Y
Clst_0899/900	998883	C	A	NA	Y
Clst_0972/3	1090847	-	A	1	Y
Clst_0992*	**1116366**	**-**	**A**	**1**	**Y**
Clst_1060*	**1201128**	**-**	**A**	**1**	**Y**
Clst_1117*	**1271246**	**AT**	**-**	**−2**	**Y**
Clst_1187	1342072	T	A	NA	Y
Clst_1286/7	1442396	-	T	1	Y
CLst_1298/9	1457047	-	T	1	Y
Clst_1339/40	1499569	A	-	−1	Y
Clst_1341	1502214	A	T	0	Y
Clst_1420/1	1596278	A	T	0	Y
Clst_1435	1606866	-	A	1	Y
Clst_1435	1606893	T	-	−1	Y
Clst_1474/5	1649400	-	T	1	Y
Clst_1511/2	1694436	A	T	0	N
Clst_1524/5	1707519	T	-	−1	N
Clst_1542/3	1724606	C	T	0	N
Clst_1543	1725709	T	C	0	Y
Clst_1605/6	1809359	-	T	1	Y
Clst_1771/3	2005558	A	T	0	N
Clst_1893	2151838	AA	GG	0	Y
Clst_2041/2	2322549	T	A	0	N
Clst_2046	2314413	-	C	1	N
Clst_2051	2322540	-	C	1	N
Clst_2090/1	2365983	G	T	0	N
Clst_2360	2656461	-	CTC	NA	N
Clst_2564/5	2854567	-	T	1	Y

^1^Indicates position in genome of corrected sequence (or former position in case of deleted base). ^2^Offset based on inserted or deleted base(s). NA = Not applied, indicates that final version of sequence was not changed based on RNA-seq (see Additional file 1). *Rows in bold indicate that RNA-seq data corroborates suggested changes by GenePRIMP/NCBI (see Additional file 2: Table S1).

### Proteogenomic confirmation of genome annotation

**Figure 2:**
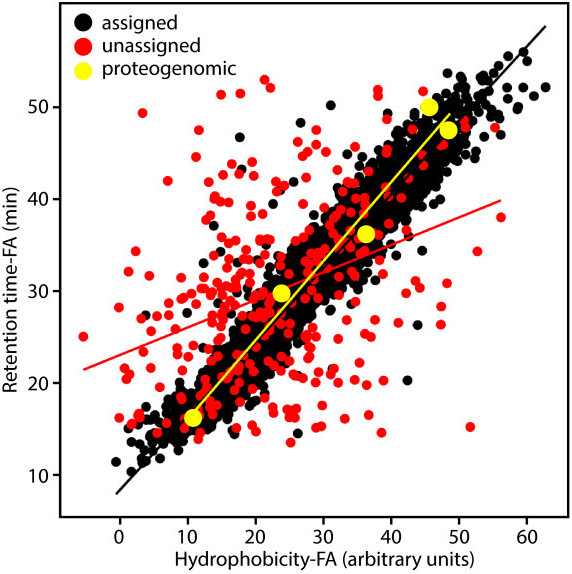
***Proteogenomic analysis of C. stercorarium DSM8532*** ^***T***^ **.** Correlation of hydrophobicity and retention time in formic acid (FA) for peptides mapped to a 6-reading frame database derived from raw 454 reads of *C. stercorarium* whole genome sequence, including peptides assigned to matching proteins of the annotated genome (black), peptides not matching annotation (red) and “proteogenomic” peptides resulting in changes to annotation (yellow).

By comparing the proteogenomic database to annotated proteins, our analysis yielded 6,611 peptides aligning with annotated proteins and 312 peptides that did not align (expectation value cutoff for all peptides log(e) < −1). The confidence of peptide sequence assignment was further strengthened by comparing computed hydrophobicity versus retention time (R^2^ = 0.93) (Figure 2). Correlation of hydrophobicity and retention time for unassigned peptides was much weaker (R^2^ = 0.15). Of this collection, only two peptides (DLAYKGQIPGVR and ICGRPHAYMR) were found in the same reading frame with nearby coordinates, indicating that they identified a missed protein in the annotation. These peptides were found to align with similar coordinates in the *C. thermocellum* DSM1237 protein A3DJI5 (30S ribosomal protein S14). One peptide (FMPELDILQK) supported an alternate reading frame that was also identified by GenePRIMP. This analysis provides observational support for annotation modifications suggested in the genome improvement workflow (Additional file 3: Table S2). Two more peptides in this collection (FLNEDLPLEER and MDMSQYLGIFVEESR) supported 5’ extensions of annotated proteins that were not suggested by GenePRIMP. Correlation of hydrophobicity/retention time for these five “proteogenomic” peptides was similar to assigned peptides (R^2^ = 0.97). Although these observations were not independently confirmed, they are corroborated by the annotation of the alternate genome for this organism [40].

### Corroboration of pseudogenes

A total of 34 genes identified as pseudogenes in the automatic annotation or through the genome improvement pipeline were found to have no peptides associated with them under the culture conditions in this study and were identified as such in the final annotated genome. Two other loci (Clst_0108 and Clst_1866) were found to have at least some peptide coverage (2% and 8% respectively) and were not annotated as pseudogenes. Most of the suggested alternatives by GenePRIMP/NCBI (76/94 or 83%) were identified as pseudogenes, however all alternatives corroborated by RNA-seq/proteogenomic analysis were not (Additional file 3: Table S2). These included three suggested gene joins (Clst_0130/1, Clst_0746/7 and Clst_0991/2) that were initially predicted to be pseudogenes, but had at least some coverage by peptides (19%, 6% and 1%, respectively) and were found to have intact reading frames once RNA-seq corrections were applied. Three other cases of suggested gene joins resulting in putative pseudogenes (Clst_0150/1, Clst_0580/1 and Clst_1874/5) were covered by peptides (0% and 5%, 0% and 2%, 45% and 5% respectively for unjoined genes) and were not joined in the final annotation. BLAST analysis was also performed in order to ensure that peptides observed for putative pseudogenes with peptide coverage were not misattributed due to presence of orthologous genes in the genome. Given inherent biases and contrasts between different gene-calling and annotation improvement algorithms, further in-depth proteomics analysis of sequenced organisms under different culture conditions will be required to test whether annotated pseudogenes are actually coding regions.

### Carbohydrate-active enzymes

A total of 106 genes encoding proteins with predicted activity on carbohydrates were identified through the CAZy database, including 67 glycoside hydrolases (GH), 18 glycosyltransferases (GT), 10 carbohydrate esterases (CE), 5 polysaccharide lyases (PL) and 8 genes with carbohydrate-binding motifs (CBM)/surface layer homology (SLH) domains only (Additional file 4: Table S3). GH enzymes from 32 different CAZy families were observed. Seventeen of these proteins, incuding 12 GH enzymes, were modular with multiple catalytic regions and/or one or more CBM/SLH domains. These results confirm 17 previously sequenced biomass-degrading enzymes identified for this organism, including cellulases (*celYZ*), cellobiose phosphorylases (*cepAB*), xylanases (*xynABC*), xylosidases (*xylAB, bxlAB, bglZ*), arabinofuranosidases (*arfAB*), a galactosidase (*agaA*), a pectate lyase (*pelA*) and an α-rhamnosidase (*ramA*) (98-100% identical to coding regions identified in genome sequence). Peptides were observed for 85/105 or 81% of genes in this category, with highest peptide coverage (45-54%) of previously described genes for ArfB, BglZ, CepB and BxlA (Additional file 4: Table S3).

### Putative ABC-type carbohydrate transporters

**Figure 3:**
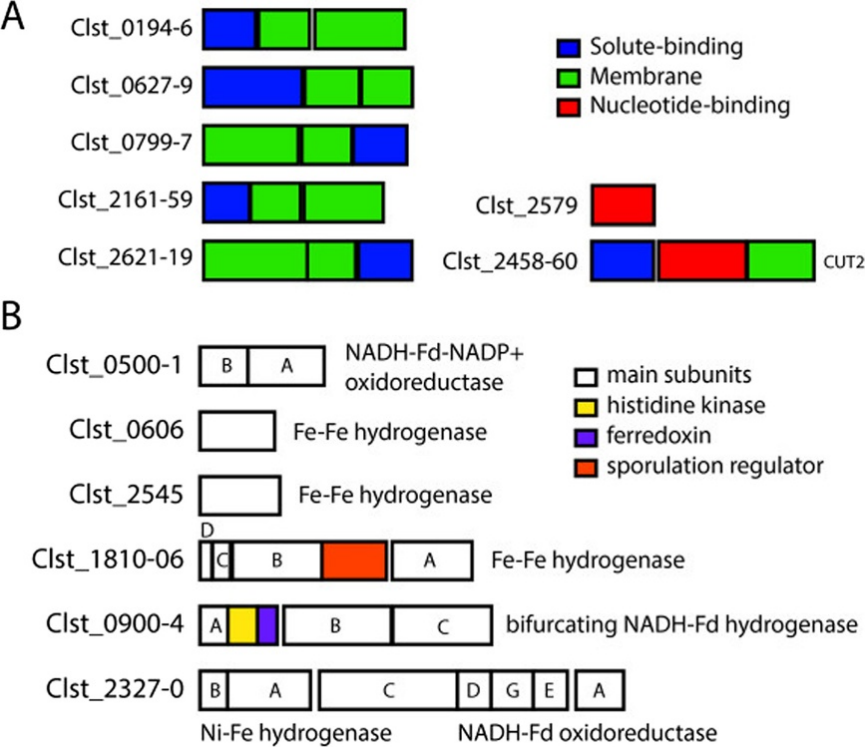
***Structure of ABC transporters and genes involved in hydrogen production and co-factor recycling.*** **A**. Seven ABC transporters for which all proteins were observed, including solute-binding (blue), membrane (green) and nucleotide-binding (red) subunits, as defined by NCBI. *Note:* Clst_2458-60 is in Carbohydrate Uptake Transporter Group 2 (CUT2) while others are CUT1. **B** *.* Main enzymatic subunits (white squares) and co-located genes (coloured squares) for six clusters designated as hydrogenases and/or oxidoreductases.

Of 372 enzymes with Transporter Classifications, 242 belong to the ATP-binding cassette (ABC) superfamily 3.A.1, and 118 of this subset are predicted carbohydrate importers based on automated annotation, organized in 42 contiguous clusters (Table 2). Although annotations of transmembrane (M) vs. solute-binding (S) enzymes were often inconsistent between IMG and ABCdb, 114 out of 118 enzymes fell into these two categories, leaving only 4 nucleotide-binding (N) enzyme-coding loci with predicted carbohydrate uptake activity (Table 2). Most of these were from Carbohydrate Uptake Transporter (CUT) family 1 (3.A.1.1), with 8/118 in 2 clusters belonging to CUT family 2 (3.A.1.2). Most CUT genes were organized in groups of 2–5 adjacently located coding regions, with all but two of these clusters containing M and S enzymes only. One cluster contained 5 genes, including 2 N, 2 M and an S enzyme (family CUT2), while four enzymes were not co-located with other CUT enzymes (1 N, 1 M and 2 S enzymes). Less than half of these proteins (54/118 or 46%) were covered by peptides under these culture conditions. Peptides were observed for every gene in only 8 clusters, suggesting these specific protein clusters may be of particular relevance for transport *in vivo* (Figure 3A, Table 2).

**Table 2:** **Organization and observation of ABC-type carbohydrate uptake transporters in genome, transcriptome and proteome of** ***C. stercorarium*** **DSM8532** ^**T**^

Cluster		Order of genes in cluster ^2^		Peptide coverage (%):
Loci range ^1^	Strand	IMG/ER	ABCdb	
Clst_0059-0061	+	S-S-M	M-M-X	0/4/11
Clst_0109-0112	+	M-M-S-S	M-M-X-S	0/0/0/0
Clst_0194-0196	+	S-S-M	S-M-M	53/10/13
Clst_0200-0202	+	S-S-M	M-M-S	0/0/2
Clst_0209-0211	+	S-M-S	S-M-M	4/0/0
Clst_0215-0217	+	S-S-M	M-M-S	0/0/37
Clst_0218-0221	+	S-S-S-M	S-S-M-M	8/16/0/0
Clst_0228-0230	+	M-M-S	M-M-S	0/0/36
Clst_0432-0434	+	M-M-S	M-M-S	0/0/10
Clst_0444-0446	−	S-M-S	S-M-M	0/0/5
Clst_0456-0460*	+	N-N-M-S-M	N-N-M-M-S	12/2/0/2/9
Clst_0472-0473	+	M-M	M-M	0/0
Clst_0476	+	S	S	0
Clst_0479-0481	+	S-M-M	S-M-M	34/0/5
Clst_0582	−	S	S	0
Clst_0627-0629	+	S-M-M	S-M-M	24/3/7
Clst_0666-0667	+	S-S	X-M	0/0
Clst_0673-0674	+	M-M	M-M	0/0
Clst_0797-0799	−	M-M-S	M-M-S	16/3/4
Clst_0805-0807	−	M-M-S	M-M-X	18/0/0
Clst_0848-0850	−	S-M-M	S-M-M	4/0/34
Clst_0934-0936	+	M-S-S	S-M-M	24/0/3
Clst_0969-0971	+	S-S-M	M-M-S	0/0/6
Clst_0993-0995	+	S-S-S	M-M-S	0/0/4
Clst_1007-1008	+	M-S	M-M	0/0
Clst_1068-1070	+	M-S-S	S-M-M	5/0/0
Clst_1073-1075	+	S-M-S	M-M-S	0/0/18
Clst_1077-1079	+	M-M-S	S-M-M	3/4/0
Clst_1083-1085	+	S-S-M	M-M-S	0/0/18
Clst_1566-1567	−	M-S	M-M	0/0
Clst_1587-1589	−	M-S-S	S-M-M	0/0/29
Clst_1635-1637	+	S-M-S	S-M-M	24/0/0
Clst_2117-2119	−	M-M-S	M-M-S	0/0/0
Clst_2139-2141	−	S-M-M	S-M-M	0/0/15
Clst_2159-2161	−	M-M-S	S-M-M	14/22/28
Clst_2245-2247	−	S-M-M	S-M-M	0/0/36
Clst_2458-2460*	+	M-N-S	S-N-M	59/69/9
Clst_2539-2541	−	S-M-S	S-M-M	28/0/0
Clst_2544	−	M	S	23
Clst_2579	−	N	N	41
Clst_2595-2597	−	S-S-M	S-M-M	0/0/43
Clst_2619-2621	−	S-M-S	M-M-X	28/4/9

^1^Range of loci in genome for genes in cluster (ie. Clst_0059-Clst_0061), ^2^Order of genes in cluster according to IMG Expert Review annotation platform (IMG/ER) or the ABC transporter database (ABCdb), S = Solute-binding protein, M = Transmembrane domain, N = nucleotide (ATP)-binding domain, X = No match in ABCdb, *Indicates that genes in cluster belong to Carbohydrate Uptake Transporter family 2 (CUT2).

### Glycolysis and pentose phosphate pathway

**Figure 4:**
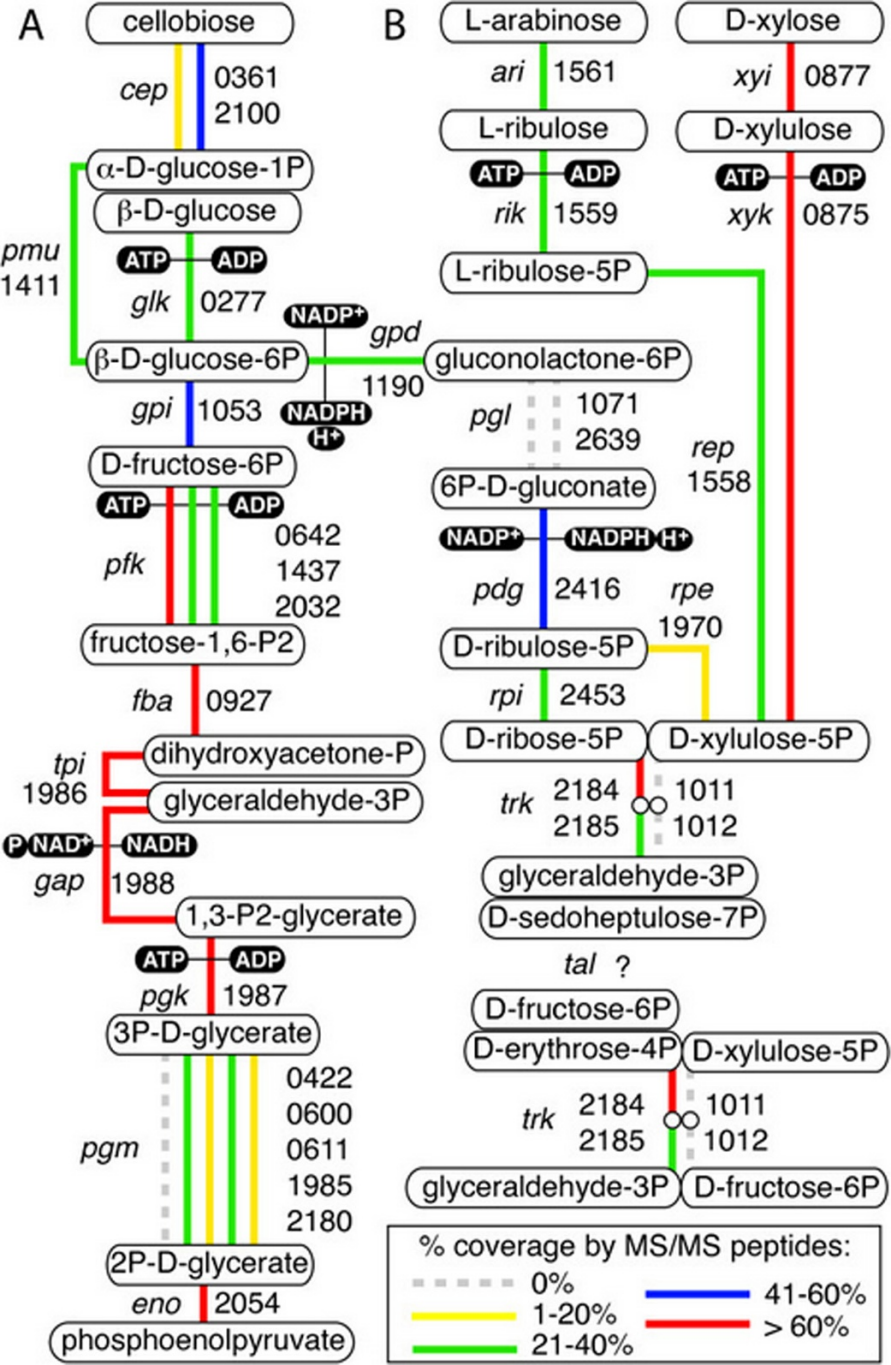
***Partial glycolytic and pentose phosphate pathway enzymes in C. stercorarium DSM8532*** ^***T***^ ***.*** Boxes show metabolic intermediates while lines indicate catalysis, with segmented lines joined by hollow circles indicating multi-enzyme complexes and parallel lines indicating enzymes with identical annotated functions (not all shown for visual clarity). Filled boxes indicate enzyme co-factors. Numbers indicate locus tag (ie. Clst_####). Colour of lines indicates percent coverage of gene by mapped peptides. **A**. Glycolysis, cellobiose/glucose to PEP, **B**. Pentose phosphate pathway, oxidative and non-oxidative. Gene symbols: *cep* = cellobiose phosphorylase, *pmu* = phosphoglucomutase, *glk* = glucokinase, *gpi* = glucose-6-phosphate isomerase, *pfk* = phosphofructokinase, *fba* = fructose bisphosphate aldolase, *tpi*, triosephosphate isomerase, *gap* = glyceraldehyde-3-phosphate dehydrogenase, *pgk* = phosphoglycerate kinase, *pgm* = phosphoglycerate mutase, *eno* = enolase, *ari* = arabinose isomerase, *rik* = ribulose kinase, *xyi* = xylose isomerase, *xyk* = xylulose kinase, *rep* = ribulose-5-phosphate 4-epimerase, *rpe* = ribulose-5-phosphate 3-epimerase, *gpd* = glucose-6-phosphate 1-dehydrogenase, *pgl* = 6-phosphogluconolactonase, *pdg* = 6-phosphogluconate dehydrogenase, *rpi* = ribose-5-phosphate isomerase B, *trk* = transketolase, *tal* = transaldolase.

With the exception of transaldolase, all genes with functional annotations associated with central glycolytic and pentose phosphate pathways were observed in the genome. Three phosphofructokinases (Pfk) were annotated and all had some coverage by peptides (Figure 4A), while only 4 out of 5 annotated phosphoglucomutases (Pgm) were observed in the proteome. Extensive coverage of xylose isomerase (Xyi) and xylose kinase (Xyk) by peptides was observed, indicating pentose utilization by this organism (Figure 4B). Only one of two annotated copies of the transketolase A and B genes were observed in the proteome (Clst_2184/5). Although glucose phosphate dehydrogenase (Gpd) converting glucose-6P to gluconolactone-6P was observed, neither gene annotated as phosphoglucolactonase (Pgl) had any associated peptides under these culture conditions, indicating a possible alternative source of 6P-D-gluconate for D-ribulose-5P synthesis. No known transaldolase (Tal) was observed in the genome or through proteogenomic analysis, indicating the existence of an alternative pathway for sedoheptulose-7P (S-7P) degradation in the pentose phosphate pathway.

### PEP/pyruvate conversion and co-factor recycling

**Figure 5:**
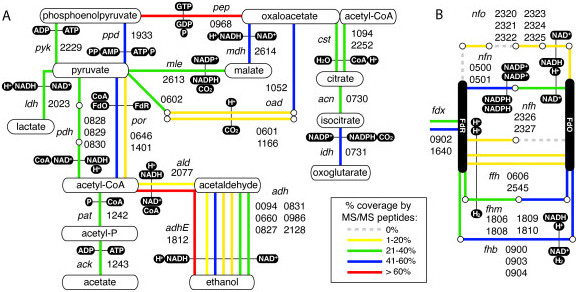
***PEP/pyruvate conversion and co-factor recycling enzymes in C. stercorarium DSM8532*** ^***T***^ ***.*** Details as for Figure 4. **A**. PEP/pyruvate conversion to lactate, acetate, ethanol or oxoglutarate. **B**. Co-factor recycling and hydrogenases. Gene symbols: *pyk* = pyruvate kinase, *ppd* = pyruvate phosphate dikinase, *ldh* = lactate dehydrogenase, *pdh* = pyruvate dehydrogenase complex, *por* = pyruvate:ferredoxin oxidoreductase, *pat* = phosphate acetyltransferase, *ack* = acetate kinase, *ald* = aldehyde dehydrognase, *adh* = alcohol dehydrogenase, *adhE* = type IV bifunctional acetaldehyde/alcohol dehydrogenase, *pep* = phosphoenolpyruvate carboxykinase, *mdh* = malate dehydrogenase, *mle* = malic enzyme, *oad* = oxaloacetate decarboxylase, *cst* = citrate synthase, *aco* = aconitase, *idh* = isocitrate dehydrogenase, *fdx* = ferredoxin, *nfo* = NADH:ferredoxin oxidoreductase, *nfn* = NADH:ferredoxin: NADP + oxidoreductase, *nfh* = nickel-iron hydrogenase, *ffh* = monomeric iron-only hydrogenase, *fhm* = multimeric iron-only hydrogenase, *fhb* = bifunctional NADH:ferredoxin hydrogenase.

Several potential pathways for PEP/pyruvate conversion were observed, including pyruvate kinase (Pyk), pyruvate dikinase (Ppd), PEP decarboxykinase (Pep) in combination with the malate dehydrogenase/malic enzyme (Mdh/Mle) shunt, or oxaloacetate (Oad) (Figure 5A). Conversion of pyruvate to acetyl-CoA may occur through two pyruvate:ferredoxin oxidoreductases (Por) or pyruvate dehydrogenase complex (Pdh), all of which were observed in the proteome. The bifunctional type IV alcohol/acetaldehyde dehydrogenase (AdhE), a single aldehyde dehydrogenase (Ald) and 6 alcohol dehydrogenases (Adh) were observed with varying levels of peptide coverage. Lactate hydrogenase (Ldh) and phosphoacetyltransferase/acetate kinase (Pat/Ack) were also observed in the genome and proteome. Oxoglutarate synthesis from oxaloacetate via citrate/isocitrate was also indicated by observation of the required enzymes in the proteome (Figure 5A).

Ferredoxin-mediated regeneration of energy intermediates and/or hydrogen production was the annotated function of 21 coding regions, 18 of which are organized in 5 clusters (Figure 5B). Three Fe-Fe hydrogenases (2 monomeric and one tetrameric), a trimeric bifunctional NADH-oxidizing hydrogenase co-located with ferredoxin (Fdx), and a newly described dimeric enzyme complex coupling reduction of NADP + with oxidation of ferredoxin and NADH [47] (illustrated in Figure 3B) were all observed in the proteome. A dimeric Ni-Fe hydrogenase and the 6-enzyme complex NADH:ferredoxin oxidoreductase (Nfo), also known as Rnf, were co-located in the genome, however only some components of these clusters were covered by peptides, indicating that they may play a less important role in metabolism under these culture conditions (Figure 5B).

### Confirmation of end products predicted through pathway analysis

**Figure 6:**
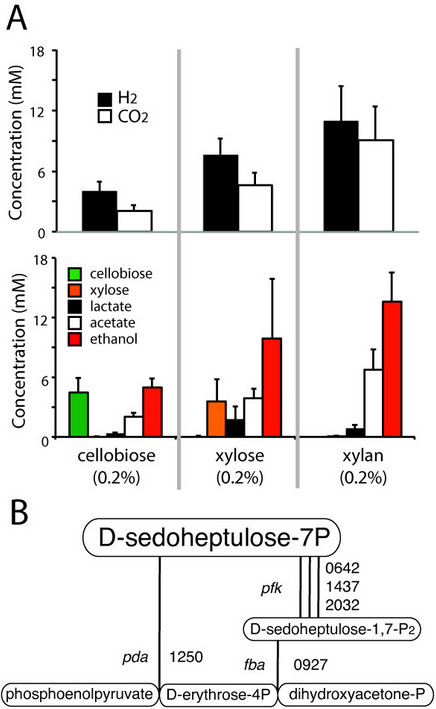
***End products of C. stercorarium DSM8532*** ^***T***^ ***.*** **A**. Gas (top) and liquid (bottom) end products on cellobiose, xylose and xylan at mid-exponential phase (12 h anaerobic culture at 60°C). Mean and standard deviation of 25 replicates. **B**. Hypothetical alternatives to transaldolase for S-7P degradation in *C. stercorarium* DSM8532^T^. S-7P may be phosphorylated by one of three phosphofructokinases (*pfk*) and subsequently cleaved to E-4P and DHA-P by fructose bisphosphate aldolase (*fba*). Alternatively, S-7P may be cleaved to PEP and E-4P by 3-deoxy-D-arabinoheptulosonate-7-phosphate synthase (*pda*), an enzyme usually active in creating an S-7P-like molecule from PEP/E-4P in the shikimate pathway.

In order to confirm function of lignocellulolytic, transport and central metabolic pathways, *C. stercorarium* DSM8532^T^ was cultured on cellobiose, xylose and xylan (purified hemicellulose) and harvested at mid-exponential phase (Figure 6A). Results confirmed growth on xylan consistent with the presence of several xylanolytic enzymes (Additional file 3: Table S2). Profile of observed end products (carbon dioxide, lactate, acetate, ethanol) is consistent with the presence of required enzymes in pyruvate conversion pathways, while detection of hydrogen in gas phase is consistent with hydrogenases and oxidoreductases detected in this organism. Growth on xylose is consistent with presence of a known xylose transporter (XylFGH) and an intact pentose phosphate pathway. Given absence of transaldolase in the genome, alternative pathways for degradation of sedoheptulose-7P (S7P) are proposed (Figure 6B).

## Discussion

We report a complete genome sequence for *C. stercorarium* DSM8532^T^, generated using a single sequencing platform and *in silico* gap-filling. This approach represents a significant advance and savings in wet-lab procedures for gap-closing, however a key requirement of the *in silico* technique is complete coverage of the genome by sequencing reads (Thallinger *et al.*, manuscript in preparation). Validation of the approach was provided by the recent release of a concurrent sequence where extensive wet-lab gap-closing was applied [40]. Although a comparison of the two versions is beyond the scope of this study, a preliminary analysis indicates very few differences between the two, mostly found in an ambiguous region of our sequence.

Genome-wide studies increasingly employ complementary omics data in order to improve annotation and provide enhanced insight into bacterial metabolism [12, 48–53]. We report extensive coverage of the genome by transcripts and peptides using updated techniques (sequence-based RNA profiling, gel-free 2D proteomics). Complete coverage of virtually all genes by RNA-seq reads may indicate a background level of transcription for the entire genome. Although DNAseI treatment of cell extracts was performed, some residual DNA was observed in the RNA prep using fluorometric assays, and may have been sequenced at a background level [54]. Therefore, further work will be required to ensure absence of DNA or subtraction of background signal. Despite this limitation, RNA-seq data were effectively applied to improve the genome sequence and correct homopolymer errors resulting from 454 sequencing, corroborating several suggestions for sequence changes proposed by the genome annotation improvement pipeline GenePRIMP and NCBI prior to sequence submission. Our results indicate that Illumina RNA-seq for WGS improvement may supplant Illumina DNA sequencing for this purpose [55], since genome coverage using RNA prepared as described in this study was sufficient to correct the majority of the homopolymer errors, even in non-coding regions.

Proteogenomic analysis resulted in several suggested improvements to the genome sequence, corroborating an inserted gene and an alternative reading frame suggested by GenePRIMP, as well as probable gene extensions not captured by other genome improvement procedures. Overall, proteogenomic analysis indicates the quality of the automated genome annotation in that only two cases of previously unannotated genes were observed, both of which were also highlighted through GenePRIMP.

Most of the genome improvements suggested by GenePRIMP and/or NCBI resulted in putative pseudogenes. However, a subset of suggestions (16/94) were not identified as pseudogenes and half of these (8/16) were corroborated by RNA-seq or proteogenomic analysis. This finding indicates that genome improvement suggestions by GenePRIMP or NCBI algorithms may be more reliable when not resulting in pseudogenes. An increased effort by bioinformaticians to identify erroneous pseudogenes in existing databases would be desirable, confirming the importance of using multiple data sources to improve genome annotation. Further, observation of peptides through proteomics provides direct insight into whether a particular coding region should or should not be defined as a pseudogene. Although only a single culture condition was used in this study, most coding regions defined as pseudogenes had no peptides observed, corroborating automatic annotation by IMG/ER, GenePRIMP and/or NCBI.

Since the purpose of this study was an improved whole genome sequence and annotation, we included all data meeting minimum quality standards to determine coverage of gene regions by RNA-seq and MS/MS proteomics data. Further work will be required to determine DNA contamination and/or background noise related to RNA-seq [54]. High-throughput proteomics of HPLC-fractionated peptides in this study has provided exceptional depth of coding region coverage, comparable to other in-depth studies [12]. However, signals generated by different proteomics and transcriptomics platforms may be biased by a number of factors [56, 57]. For example, samples are from cultures growing on hexose (transcriptome) or pentose (proteome), and cannot be strictly compared due to undetermined effects on the regulatory milieu, gene expression, relative abundance of transporters and branching central metabolic pathways more generally [12]. Further work will be required to determine the relation of these terms to cellular metabolism and each other as technologies to measure them continue to evolve [16, 58, 59].

Lignocellulolytic enzymes are the only previously well-characterized components of *C. stercorarium* DSM8532^T^ metabolism. In this study, some of the most important predicted cellulases were not detected in the proteome. Many lignocellulolytic enzymes encode transmembrane domains (25/106 or 24%), signal peptides (29/106 or 28%) or both (20/106 or 19%). Since proteomic profiles were generated from cell pellets growing on soluble sugars, detection of this enzyme class may be limited due to presence in culture supernatants only or possible lack of expression in the absence of lignocellulosic material [12]. Further studies on complex substrates such as cellulose or hemicellulose will be required to determine their relative importance in lignocelluloysis. Although many of this organism’s nearest phylogenetic (16S) neighbours are cellulosome-encoders, CelYZ of *C. stercorarium* most closely resembles a similar pair in *C. phytofermentans*, another cellulolytic organism without cellulosome. Homologous enzymes containing cellulosomal dockerin domains are grouped together with another cellulase and adjoining cellulolosomal scaffoldin in *C. papyrosolvens* and *C. cellulolyticum*, while homologs are also present as non-dockerin-containing enzymes in *C. thermocellum*, although vastly separated from each other on the chromosome. These observations indicate the complex interweaving and reiteration of coding sequences across phylogenetically close but functionally divergent organisms, providing insight into horizontal gene transfer and small replicon-mediated evolution.

All but one ABC-type CUT cluster contained transmembrane and solute-binding components only, an arrangement frequently observed in Gram-positive organisms [60]. We focused on a subset of 7 CUT clusters where every gene in the cluster was detected by MS/MS. Most clusters consisted of 2 transmembrane genes (COG0395 and COG1175) and a single solute-binding gene (COG4209) with clostridial homologues in *C. termitidis*, *C. phytofermentans*, *C. papyrosolvens* and *Thermoanaerobacterium xylanolyticum*. Surprisingly, several genes in these clusters, including Clst_0194-6, had near neighbours in *Treponema* (50-60% identical at amino acid-level), an otherwise distantly-related spirochaete. The nearest homologue for the nucleotide-binding gene was from *C. thermocellum* (78% identical). The single CUT2 cluster was homologous to the known xylose transporter XylFGH of *Thermoanaerobacter pseudethanolicus* and the newly described *T. thermohydrosulfuricus* WC1 [17].

Xylose transport and utilization by this organism confirms the importance of the pentose phosphate pathway, however lack of a known encoded transaldolase (EC 2.2.1.2, EC 4.1.2.-), indicates an alternative pathway for S-7P degradation. Two possibilities are proposed, the first involving one of three encoded phosphofructokinases (Pfk) and a bifunctional fructose bisphosphate aldolase (Fba) [61] (Figure 6B). Recently, “forcing” this degradation in a transaldolase-knockout strain of *E. coli* may have occurred due to accumulation of S-7P, followed by creation of sedoheptulose 1,7-2P and cleavage to dihydroxyacetone-P (DHA-P) and erythrose-4P (E-4P) by Pfk and Fba respectively [62]. Of 345 clostridia with whole genomes in IMG, 62 (18%) do not have an annotated transaldolase, however few previous studies have shown xylose utilization by confirmed transaldolase-deficient strains [63]. Annotated Pfk genes may have distinct biological roles and use either pyrophosphate or ATP as a phosphate donor [64], therefore, we hypothesize that one of three Pfk may be involved specifically in transformation of S-7P to S-1,7-2P. An alternative hypothesis is that expression of 3-deoxy-D-arabinoheptulosonate-7-phosphate synthase (Pda) from the shikimate pathway creates a S-7P-like molecule from PEP and glyceraldehyde-3P (G-3P) and might catalyze the degradation of S-7P to PEP and G-3P during xylose utilization (Figure 6B). Further work will be required to define these potentially significant observations for pentose utilization in clostridia.

Muliple pathways for PEP/pyruvate conversion and co-factor recycling are consistent with previous literature on thermophilic clostridia [10] and demonstrated end products for this organism during anaerobic culture. All expressed clusters are likely to contribute to hydrogen production and NADH oxidation, including a bifurcating NADH-Fd hydrogenase (Clst_0900, Clst_0902-4) [65] with similarity to the designated bifurcating hydrogenase in *C. thermocellum* ATCC 27405, and a dimeric three-way oxidoreductase involving NADH, ferredoxin and NADP (Clst_0500-1) [47]. Further study will be required to determine which pathways are most highly expressed and how they are regulated during metabolism, resulting in observed end product profiles for *C. stercorarium* on cellobiose, xylose and xylan. These profiles largely confirm previous culture-based literature [4, 20, 66, 67], including generally elevated production of ethanol relative to lactate and acetate. Further testing of the robustness and reproducibility of molecular techniques in relation to culture-based parameters will help to determine a theoretical baseline of expected values for mid-exponential cells, linking expression profiles to strain performance in terms of efficient substrate utilization and ethanol production.

## Conclusions

We report a finished WGS for this well-characterized type strain in the context of detailed information about coverage of annotated gene regions using Illumina RNA-seq and high-throughput 2D MS/MS. To our knowledge, this is the first time a WGS has been enhanced using these advanced techniques. Our approach may represent an updated model for better definition of the molecular systems biology of an organism in an era where WGS have proliferated rapidly. Understanding the influence of environmental factors on expression of inter-connected enzymatic pathways will be critical to evaluate and improve ethanol production by selected organisms and consortia in consolidated bioprocessing.

## Electronic supplementary material

Additional file 1: **Custom Scripts.** (DOCX 64 KB)

Additional file 2: Table S1: Summary of gene regions in *C. stercorarium* DSM8532^T^ and coverage by RNA-seq reads and MS/MS peptides. (TXT 172 KB)

Additional file 3: Table S2: *C. stercorarium* DSM8532^T^ genome sequence edits suggested by GenePRIMP/NCBI. (TXT 8 KB)

Additional file 4: Table S3: Annotation of carbohydrate-active enzymes in *C. stercorarium* DSM8532^T^ and coverage by MS/MS peptides. (TXT 6 KB)
