# Supplementary material for: Enhanced whole genome sequence and annotation of Clostridium stercorarium DSM8532T using RNA-seq transcriptomics and high-throughput proteomics
Source: BMC Genomics. 2014 Jul 7;15(1):567. doi: 10.1186/1471-2164-15-567 (PMC4102724; doi:10.1186/1471-2164-15-567)
Supplement: Supplementary file 1 — Additional file 1: Custom Scripts. (DOCX 64 KB) [file 12864_2013_6261_MOESM1_ESM.docx]

**Additional File 1**

**Supplementary Text: Custom Scripts**

CUSTOM SCRIPTS:

**1. adapter.py and qsub shell script** (p. 2-4)

**2. direct_bpmapper.py** (p. 5-11)

**3. bpmconvert.R** (p. 12-15)

**1. adapter.py and qsub shell script** (X. Jiang)

######

# Python script searching adapter sequences in raw reads

# adapter.py

# Author: Xiangli Zhang

# Email: [zhangju@cc.umanitoba.ca](mailto:zhangju@cc.umanitoba.ca) & [dlyzxl@yahoo.com](mailto:dlyzxl@yahoo.com)

*###*

*#Pipeline qsub shell script of RNASeq reads preprocessing and*

*#*expression analysis with Tophat/Cufflinks

###

#!/bin/bash

#PBS -S /bin/bash

#PBS -l nodes=1:ppn=24,mem=25gb

#PBS -l walltime=72:00:00

#PBS -m bea

#PBS -M [zhangju@cc.umanitoba.ca](mailto:zhangju@cc.umanitoba.ca)

#The above is qsub script requesting computational resources from Westgrid cluster

app=/home/zhangju/App

#Adding paths for Bowtie2/Tophat/Cufflinks/Samtools

export PATH=$PATH:$app/bowtie2-2.0.2:$app/cufflinks-2.0.2.Linux_x86_64:$app/samtools-0.1.18:$app/tophat-2.0.6.Linux_x86_64

#number of processors requested by qsub

NUM_PROCS=`/bin/awk 'END {print NR}' $PBS_NODEFILE`

#base directory for each genome with multiple samples

base=/global/scratch/zhangju/RNASeq_2013/Analysis/Cstercorarium

cd $base

genome=`basename $base`

#get genbank file of the genome

genbank_extension="gbf"

s1=`ls index/*.$genbank_extension`

genbank=$base/$(echo $s1 | cut -d ' ' -f 1)

genome_fasta="$genbank"_genome.fna

#extract fasta sequence from genbank file

/home/zhangju/bin/perl /home/zhangju/App/genbank2fasta.pl $genbank

cp genome_fasta "$genbank"_genome.fa

#create bowtie2 index of genome fasta sequence

BINDEX="$genbank"_genome

bowtie2-build $genome_fasta $BINDEX

#generate GFF3 file with genbank file using bioperl script

perl $app/bp_genbank2gff3.pl -o $base/index/ $genbank

gtf_file=$genbank.gff

#generate transcript fasta file with Tophat tool gtf_to_fasta

transcript_fasta="$genbank"_transcript.fa

gtf_to_fasta $gtf_file $genome_fasta $transcript_fasta

TRANSINDEX="$transcript_fasta"_transcript

#build transcript bowtie2 index

bowtie2-build $transcript_fasta $TRANSINDEX

GTF=$gtf_file

#Looping to analyse all samples

for sample_dir in $(ls -d sample*/);

do

sample1=${sample_dir//\//$'\n'}

sample=$(echo $sample1 | sed 's/ //g')

cd $sample

$suffix=$sample

fq_file=$(echo $sample | cut -d ' _' -f 2)

#Illumina PE fastq files

fastq_extension="fastq.gz"

s1=`ls *.$fastq_extension`

#Input fastq files to trimmomatic for preprocessing

R1_raw=$(echo $s1 | cut -d ' ' -f 1)

R2_raw=$(echo $s1 | cut -d ' ' -f 2)

#Output files after trimmomatic preprocessing

#files of paired reads

R1=lane1_forward_paired.fq.gz

R2=lane1_reverse_paired.fq.gz

#files of unpaired reads

R1_unpaired=lane1_forward_unpaired.fq.gz

R2_unpaired=lane1_reverse_unpaired.fq.gz

#Subsetting enough reads for searching Illumina adapters with custom Python script adapter.py

zcat $R1_raw | head -n 80000 > test.fq

zcat $R2_raw | head -n 80000 >> test.fq

#search Illumina adapters in raw reads, and save adapter sequences in file illluminaClipping.fa

#All index sequences from Illumina Customer Sequence Letter

cp $app/illuminaClipping.fa_original ./

touch illuminaClipping.fa

python $app/adapter.py

#preprocessing raw reads with Trimmomatic tool

java -classpath $app/Trimmomatic-0.22/trimmomatic-0.22.jar org.usadellab.trimmomatic.TrimmomaticPE -threads ${NUM_PROCS} -phred33 -trimlog trim_log.txt $R1_raw $R2_raw $R1 $R1_unpaired $R2 $R2_unpaired ILLUMINACLIP:illuminaClipping.fa:2:40:15 LEADING:3 TRAILING:3 SLIDINGWINDOW:4:15

Unpaired=unpaired.fq.gz

cat $R1_unpaired $R2_unpaired > $Unpaired

rm test.fq

#Tophat first run with paired reads

Tophat_PairedOUTPUT=$base/$sample/tophat_paired_output

Tophat_UnpairedOUTPUT=$base/$sample/tophat_unpaired_output

APP="nice tophat2 -p ${NUM_PROCS} -r 300 --mate-std-dev 50 -o $Tophat_PairedOUTPUT -G $GTF --transcriptome-index=$TRANSINDEX $BINDEX $R1,$R2"

$APP

bam_file1=$Tophat_PairedOUTPUT/accepted_hits.bam

#convert bed file from paired reads to a junction for unpaired reads

bed_to_juncs < $Tophat_PairedOUTPUT/junctions.bed > $Tophat_PairedOUTPUT/paired_list.juncs

#Tophat second run with unpaired reads

APP="nice tophat2 -j $Tophat_PairedOUTPUT/paired_list.juncs -p ${NUM_PROCS} -r 300 --mate-std-dev 50 -o $Tophat_UnpairedOUTPUT -G $GTF --transcriptome-index=$TRANSINDEX $BINDEX $Unpaired"

$APP

bam_file2=$Tophat_UnpairedOUTPUT/accepted_hits.bam

#merge bam files from paired end reads and unpaired reads

merge_bam=$base/$sample/merge_bam_dir

mkdir $merge_bam

samtools sort $bam_file1 $merge_bam/paired.sort

samtools sort $bam_file2 $merge_bam/unpaired.sort

samtools merge -f $merge_bam/total.bam $merge_bam/paired.sort.bam $merge_bam/unpaired.sort.bam

# Cufflinks calculate transcript expression level FPKM value

Cufflinks_OUTPUT=$base/$sample/cufflink_merged_bam

bam_file3=$merge_bam/total.bam

APP="nice cufflinks --max-bundle-frags 4000000 -b $transcript_fasta -u -o $Cufflinks_OUTPUT -G $GTF -p ${NUM_PROCS} -v $bam_file3"

$APP

cd $base

done

**2. direct_bpmapper.py** (G. Alvare)

#!/usr/bin/env python

# TIC File Base Pair Mapper

# -------------------------

# SYNOPSIS

# Reads in a protein TIC file and converts it to a base pair map file.

#

# USAGE

# direct_bpmapper.py <genome.gbk> <tic_files.txt>

#

# Examples:

# direct_bpmapper.py e_coli_k12.gb e_coli_k12_lac-tic.txt

# direct_bpmapper.py e_coli_k12.gb e_coli_k12_gluc-tic.txt e_coli_k12_lac-tic.txt

#

# BASE PAIR MAPS

# A base pair map is specified as a 2 column TSV file (tab separated

# values). The two columns are: (1) base pair number, from the start

# of the genome; and (2) the expression value for that base pair number.

#

# LOCUS TAG MAPS

# A locus tag map is specified as a 2 column TSV file (tab separated

# values). The two columns are: (1) a locus tag; and (2) the expression

# value for that locus tag.

#

# TIC FILE

# The TIC files are space-delimited spreadsheets. These files contain

# many fields; however this program is only interested in the first and

# eigth columns. The eigth column contains a peptide, and the first field

# contains the TIC value measured for the peptide.

#

# INPUT

# This accepts any number of parameters greater than two. The first parameter

# is the genome GenBANK file containing all of the locus tag and feature

# information. All subsequent parameters are TIC files, used to determine

# base pair map expression levels from. All TIC files MUST end with: "-tic.txt".

#

# PREREQUISITES

# This program requires the following modules (version numbers in parenthesis

# are the version numbers I used, NOT the minimum or maximum version numbers):

#

# Python (2.6.1)

# Biopython (1.59 - this includes SeqIO)

#

# OUTPUT

# This program writes the output base pair maps to the directory: ./new-bpmaps/

#

###################################

# THIS IS OPEN SOURCE CODE (YAY!) #

###################################

# LICENSE

# This code is licensed under the Creative Commons 3.0

# Attribution + ShareAlike license - for details see:

# http://creativecommons.org/licenses/by-sa/3.0/

#

# PROGRAM AUTHOR

# Graham Alvare - home.cc.umanitoba.ca/~alvare

#

# ACKNOWLEDGEMENTS/CO-AUTHORS (FOR THE PROGRAM)

# Dr. Brian Fristensky - my work supervisor, and the man who introduced

# me to the wonderful field of bioinformatics.

# John Schellendberg - for asking me to help him with base pair map files

# Justin Zhang - for creating the first base pair map file w/TopHat

# Vic Spicer - for generating the TIC data to analyze

# MGCB2 project - for providing me with the funding to build this script

# Manitoba Government (Innovation, Energy and Mines) - MGCB2 co-funding

# Richard Sparling and David Levin - MGCB2 Project leads

#

# QUESTIONS & COMMENTS

# If you have any questions, please contact me: alvare@cc.umanitoba.ca

# I usually get back to people within 1-2 weekdays (weekends, I am slower)

#

# P.S. please also let me know of any bugs, or if you have any suggestions

# I am generally happy to help create new tools, or modify my existing

# tools to make them more useful.

#

# Happy usage!

import sys, os, os.path, csv, math, collections

from multiprocessing import Process, Value, Array, Manager, Lock, Pool

from Bio import GenBank

from Bio import SeqIO

#######

# Displays the current progress (as a terminal/text progress bar)

#######

# PARAMETERS

# 1. text - the current process the program is performing

# 2. progress - the percentage progress of the current process

#######

def update_progress(text, progress):

sys.stdout.write('\r{0:30s} [ {1:10s} ] {2}% ({3}/{4}) '.format(text, '#'*(progress/10), progress))

#######

# Read in the locus tag feature data from the genome GenBANK file.

#######

# PARAMETER

# the filename of the GenBANK file to read locus tag features from

# RETURNS

# 1. length of the genome

# 2. a list of all CDS sequences in the genome

# 3. a list of invalid features found in the GenBANK file

# CDS SEQUENCE SPECIFICATION

# strand - (+/-) from the GenBANK file

# start - the start position of the CDS

# end - the end position of the CDS

# protein - the peptide sequence produced from CDS translation

# locus_tag - the locus tag name of the CDS

#######

def read_gb (filename):

# define the CDS object

seq_cds = collections.namedtuple('SequenceCDS', ['strand', 'start', 'protein', 'locus_tag'])

# initialize the return objects

max_index = 0

gb_cds_list = list()

invalid_features = list()

# open the GenBANK file

handle = open(filename, 'rU')

gb_parser = SeqIO.parse(handle, "genbank")

# iterate through each record in the file

for record in gb_parser:

ckey = record.name

# read each feature in each record

for feature in record.features:

if feature.type == 'CDS':

# ensure that there is only one locus tag for the GenBank feature

if 'translation' in feature.qualifiers \

and len(feature.qualifiers['translation']) == 1:

# create a new CDS object for the CDS read in

strand = feature.location.strand

start = feature.location.nofuzzy_start + 1

end = feature.location.nofuzzy_end + 1

protein = feature.extract(record.seq).translate(table=11, cds=True).upper()

ltag = feature.qualifiers['locus_tag'][0]

gb_cds_list.append(seq_cds(strand, start, protein, ltag))

else:

# track invalid features

invalid_features.append(feature)

# increase the maximum base pair index counter, if applicable

max_index = max(len(record.seq), max_index)

# close the GenBANK file

handle.close()

return (max_index, gb_cds_list, invalid_features)

#######

# Reads in all of the data from a TIC file

#######

# PARAMETER

# the filename of the TIC file to read

# RETURNS

# a dict object with peptides as keys and expression

# values as the dict's values.

#######

def read_tic(filename):

# initialize the output dict structure

tic_data = dict()

# open the TIC file

print "Reading TIC file: " + filename

handle = open(filename, 'rU')

# read through the lines of the TIC file

for line in handle:

# extract the first and eighth columns

lsplit = line.split(" ")

if len(lsplit) > 7:

# the peptide is the eighth column

peptide = lsplit[7].upper()

# the expression value is the first column

# initialize the dict index, if necessary

if peptide in tic_data:

tic_data[peptide] += float(lsplit[0])

else:

tic_data[peptide] = float(lsplit[0])

handle.close()

return tic_data

#######

# Determines the base pair map data for a given TIC file's data

#######

# PARAMETERS

# 1. tic_records - the records contained in the TIC file

# 2. gb_records - a list of all CDS sequences in the genome

# 3. fnum - the number of the current file being processed

# 4. fmax - the total number of files to process

# RETURNS

# the base pair map count data corresponding to the TIC file read

# CDS SEQUENCE SPECIFICATION

# strand - (+/-) from the GenBANK file

# start - the start position of the CDS

# end - the end position of the CDS

# protein - the peptide sequence produced from CDS translation

# locus_tag - the locus tag name of the CDS

#######

def search_tic(tic_records, gb_records, fnum, fmax):

# initialize the data structures and progress bar

counts = dict()

gb_index = 1

istep = round(len(gb_records) / 100)

update_progress(" Processing data", 0)

for rec in gb_records:

# read all of the data from the current GenBANK CDS record

start = rec.start

protein = str(rec.protein)

ltag = rec.locus_tag

max_len = len(protein)

# search for peptides which correspond to the current

# GenBANK CDS record, and add their expression values

for peptide in tic_records:

pindex = 0

while pindex >= 0 and pindex < max_len:

pindex = protein.find(peptide, pindex)

if pindex == -1:

break

else:

# set the peptide start and end

# base pair indices

pstart = start + (pindex * 3)

pend = pstart + (len(peptide) * 3)

# copy the expression data to the

# output bpmap data structure

for index in range(pstart, pend):

if not counts.has_key(index):

counts[index] = 0

counts[index] += tic_records[peptide]

pindex += len(peptide)

# update the progress every 'istep' genbank CDS records

if gb_index % istep == 0:

update_progress(" Processing data", int(gb_index / istep))

gb_index += 1

# finalize the progress bar

update_progress(" Processing data", 100)

sys.stdout.write('\n')

return counts

#######

# Writes the base pair map data to the output file.

#######

# PARAMETERS

# 1. filename - the name of the current file to process

# 2. max_index - the maximum index in the base pair map file

# 3. counts - a dict object containing all of the base pair map data

# 4. fnum - the number of the current file being processed

# 5. fmax - the total number of files to process

#######

def write_bpmap(filename, max_index, counts, fnum, fmax):

count = 0

sum = 0

outhandle = open(filename, 'wb')

csv_out = csv.writer(outhandle, dialect='excel-tab')

# progress bar

istep = round(max_index / 100)

update_progress(" Writing: " + filename, 0)

# iterate through the base pairs and

# write the data to the output file

for index in range(1, max_index + 1):

# if there is no expression data for

# the current index, print zero

if not counts.has_key(index):

value = 0.

else:

value = counts[index]

# write the output to the file

csv_out.writerow([index, value])

# update the progress every 'istep' base pairs

if index % istep == 0:

update_progress(" Writing: " + filename, int(index / istep))

# close the output file and finalize the progress bar

outhandle.close()

update_progress(" Writing: " + filename, 100)

sys.stdout.write('\n')

#######

# the main body of the code!

if __name__=="__main__":

if len(sys.argv) > 2:

fnum = 1

fmax = len(sys.argv) - 2

gb_filename = sys.argv[1]

# Read in the GenBANK file

print "Reading GenBANK file: " + gb_filename

max_index, gb_records, invalid_features = read_gb(gb_filename)

print " ***NUMBER OF INVALID FEATURES: " + str(len(invalid_features))

# Read in all of the TIC files

for tic_filename in sys.argv[2:]:

# read in the TIC data

outname = 'new-bpmaps/' + os.path.basename(tic_filename).replace("-tic.txt", ".bpmap")

tic_data = read_tic(tic_filename)

# process the TIC data, and generate the output bpmap file.

counts = search_tic(tic_data, gb_records, fnum, fmax)

write_bpmap(outname, max_index, counts, fnum, fmax)

# increment the file counter

fnum += 1

print "DONE!"

else:

# if insufficient parameters are specified, write a help message

print "Usage: direct_bpmapper.py <genome.gbk> <tic_files.txt>"

print "Writes output to the directory ./new-bpmaps/"

**3. bpmconvert.R** (J. Schellenberg)

# Goal: To convert base pair maps derived from raw RNA-seq output

# (transcriptome) and raw MS/MS output (proteome) into: 1) .fasta

# files that summarize coverage of whole genome sequence by these

# outputs, suitable for import into Gview genome visualization

# environment, and 2) a dataframe that summarizes coverage of genes

# as defined by 5'/3' positions in the whole genome annotation.

# Inputs:

# 1) seq.txt - .fasta format of C. stercorarium WGS GenBank

# accession #CP003992 as block of 2,970,010 characters (vector of

# 42429 strings with 70 characters each, except last string with 50 # characters only), with column name "V1".

# 2) loci.txt - Data frame summarizing annotation information from

# WGS.

# Column #1 = locus: Locus tag (Clst_XXXX)

# Column #2 = g5: 5' position of gene

# Column #3 = g3: 3' position of gene

# Column #4 = glength: length of gene (ie. g3-g5+1)

# Column #5 = product: annotated identity of gene

# 3) t.txt - Transcriptome base pair map derived from bam2depth

# command in samtools

# Column #1 = position in genome 1:2970010

# Column #2 = # of RNA-seq reads in .bam file associated with

# each position

# 4) p.txt - Proteome base pair map derived from protein TIC file

# using python script directbpmapper.py

# Column #1 = position in genome 1:2970010

# Column #2 = ion current of MS/MS peptides associated with

# each position

seq <- read.delim("~/seq.txt")

loci <- read.delim("~/loci.txt")

t <- read.delim("~/t.txt")

p <- read.delim("~/p.txt")

# Step #1: Split seq into a single column with 2970010 rows.

# Step #1.1: Create a dummy df to receive the data.

a <- data.frame(matrix(NA, nrow = 42429, ncol = 70))

# Step #1.2: Define function to split characters of each string

m <- function(x) {

substr(seq$V1,x,x)

}

# Step #1.3: Define for-loop to apply function to the entire string # and dump to dummy dataframe (a)

for (x in c(1:70)) {

m(x) -> a[x]

}

# Step #1.4: Turn dataframe (a) into transposed vector (b), then

# trim off empty values

as.vector(t(a)) -> b

b <- b[-c(2970011:2970030)]

# Step #1.5: Combine b with t and p to create new dataframe (e),

# with order: 1) bp, 2) b, 3) tr, 4) pr

p$pr -> pr

cbind(t,pr,b) -> e

e <- e[,c("bp","b","tr","pr")]

# Step #2: Define new vectors of length=2970010, where each element is

# EITHER the corresponding base at that position in b (A, C, T or G) # if tr or pr at that position is greater than 0 (ie. base is

# covered), OR a dash "-" when tr or pr at that position is 0

# (ie. base is not covered)

# Step #1.1: Create dummy vectors of length=2970010 to dump data

tmap <- c(rep(NA,2970010))

pmap <- c(rep(NA,2970010))

# Step #2.2: Create functions to assign "-" at position x if tr/pr

# equals 0, or assign b[x] at position x if tr/pr is greater than 0

n <- function(x) {

if (e$tr[x] == 0) {

c("-")

} else if (e$tr[x] > 0) {

as.character(e$b[x])

}

}

p <- function(x) {

if (e$pr[x] == 0) {

c("-")

} else if (e$pr[x] > 0) {

as.character(e$b[x])

}

}

# Step #2.3: Create for-loop to repeat functions for every base pair # x and dump to dummy vectors - Note: Takes a few minutes to run

for (x in c(1:2970010)) {

n(x) -> tmap[x]

p(x) -> pmap[x]

}

# Step #2.4: Combine new vectors tmap and pmap with dataframe e, to

# create new dataframe (f)

cbind(e,tmap,pmap) -> f

# Step #3: Export transcriptomic/proteomic coverage data (f) to fasta # format that can be inputted into Gview.

# Step #3.1: Create dummy vectors to dump concatenated data

v = c(rep(NA, 2641))

w = c(rep(NA, 2641))

# Step #3.2: Create functions to concatenate .fasta headers

# (>Clst_XXXX) and elements in pmap/tmap vectors to strings using

# c2s function in seqinr package, separated by "~"

library(seqinr)

g <- function(x) {

c(">",as.character(loci$locus[x]),"~",c2s(f$tmap[loci$g5[x]:loci$g3[x]])) -> q

c2s(q)

}

h <- function(x) {

c(">",as.character(loci$locus[x]),"~",c2s(f$pmap[loci$g5[x]:loci$g3[x]])) -> r

c2s(r)

}

# Step #3.3: Create for-loop to repeat functions for each locus and

# dump to dummy vectors, then export as .txt files to home directory # for further modification

# using find/replace in Word, Excel or text editor (remove ", change

# ~ to \p)

for (x in c(1:2641)) {

g(x) -> v[x]

h(x) -> w[x]

}

write.table(v,"tfasta.txt",row.names=FALSE)

write.table(w,"pfasta.txt",row.names=FALSE)

# Step #4: Calculate number of zeros for each gene in order and

# determine proportion of coverage of each gene by tr/pr

# Step #4.1: Create dummy dataframe to dump zeros data

s <- data.frame(rep(NA, 2641),NA,NA)

# Step #4.2: Define a function to create a vector containing locus

# tag, number of zeros in tr for that gene, and number of zeros in

# pr for that gene

t <- function(x) {

as.character(loci$locus[x]) -> loc

sum(e$tr[loci$g5[x]:loci$g3[x]]==0) -> tz

sum(e$pr[loci$g5[x]:loci$g3[x]]==0) -> pz

y <- c(loc,tz,pz)

}

# Step #4.3: Define a loop to repeat function for each locus and

# dump to (s), then rename columns

for (x in c(1:2641)) {

t(x) -> u

s[x,] <- u

}

names(s) <- c("locus","zt","zp")

# Step #4.4: Create dummy vectors to dump concatenated data

pzt = c(rep(NA, 2641))

pzp = c(rep(NA, 2641))

# Step #4.5: Define functions to calculate proportion of zeros in

# each gene, subtracting zerot and zerop from glength, then dividing # by glength for that gene

zet <- function(x) {

(loci$glength[x] - as.integer(s$zt[x])) / loci$glength[x]

}

zep <- function(x) {

(loci$glength[x] - as.integer(s$zp[x])) / loci$glength[x]

}

# Step #4.5: Create for-loop to apply functions to each gene

for (x in c(1:2641)) {

zet(x) -> pzt[x]

zep(x) -> pzp[x]

}

# Step #4.6: Add vectors (d) and (h) to (s), creating dataframe (j)

# and export to .txt file

cbind(s,pzt,pzp) -> j

write.table(j,"zeros.txt",sep="\t",row.names=FALSE)
